# Quality assurance of TomoDirect treatment plans using I’mRT MatriXX

**DOI:** 10.2349/biij.8.2.e14

**Published:** 2012-04-01

**Authors:** CW Kong, SK Yu, KY Cheung, H Geng, YW Ho, WW Lam, WK Wong

**Affiliations:** Hong Kong Sanatorium and Hospital, Happy Valley, Hong Kong, China SAR.

## Abstract

**Purpose:**

To evaluate the performance of 2D-array I’mRT MatriXX for dose verification of TomoDirect treatment plans.

**Methods:**

In this study, a 2D-array ion chamber device – the I’mRT MatriXX and Multicube Phantom from IBA – was used for dose verification of different TomoDirect plans. Pre-treatment megavoltage computed tomography (MVCT) was performed on the phantom setup for position correction. After the irradiation of treatment plans on the I’mRT MatriXX and Multicube Phantom, the measured doses of coronal planes were compared with those from the planning calculations for verification. The results were evaluated by comparing the absolute dose difference in the high dose region as well as the gamma analysis of the 2D-dose distributions on the coronal plane. The comparison was then repeated with the measured dose corrected for angular dependence of the MatriXX.

**Results:**

When angular dependence is taken into account, the passing rate of gamma analysis is over 90% for all measurements using the MatriXX. If there is no angular dependence correction, the passing rate of gamma analysis worsens for treatment plans with dose contribution from the rear. The passing rate can be as low as 53.55% in extreme cases, i.e. where all doses in the treatment plan are delivered from the rear.

**Conclusion:**

It is important to correct the measured dose for angular dependence when verifying TomoDirect treatment plans using the MatriXX. If left uncorrected, a large dose discrepancy may be introduced to the verification results.

## Introduction

TomoTherapy introduced its latest HD radiation therapy platform, which allows both TomoHelical delivery mode [1–5] and TomoDirect mode. TomoDirect is a non-rotational treatment option [6] in which the radiation dose can be delivered at different discrete gantry angles with continuous couch and multileaf collimator (MLC) movement. Due to the dynamic nature of TomoDirect treatment, it is very important for medical physicists to use the right tools to achieve quality assurance goals when employing this treatment technique.

The I’mRT MatriXX detector array is widely used for routine linac MLC quality assurance (QA) [7] or IMRT validation with actual beam incidence when the gantry sensor is employed for correcting directional dependence of the detector response [8, 15]. Without applying any correction factors for angular dependence, the I’mRT MatriXX detector is still capable of producing good verification measurements for different treatments such as IMRT [9–10, 12]; RapidArc treatment [11–13]; as well as Tomotherapy head and neck helical treatment [14–15] as the directional dependence is diluted due to the averaging effect from all gantry angles [16]. In this article, the suitability of the I’mRT MatriXX detector array for pre-verification of TomoDirect treatment plan is assessed.

## Materials and Methods

**Figure 1:**
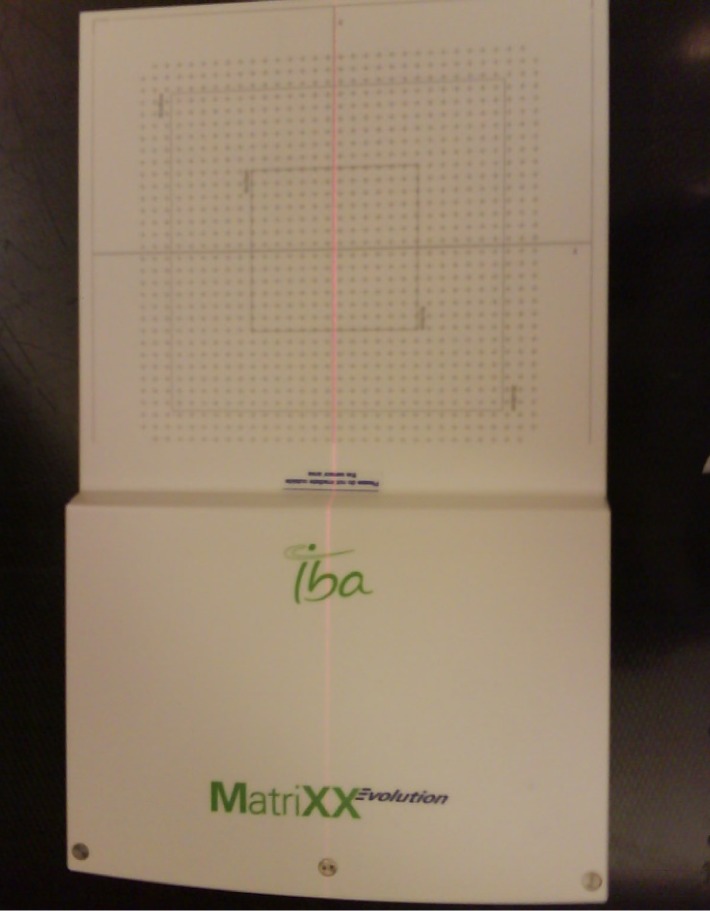
I’mRT MatriXX 2D-ion chamber array.

The I’mRT MatriXX 2D-ion chamber array (IBA Dosimetry, Schwarzenbruck, Germany) consists of 1020 vented ionization chambers (Figure 1), which are automatically corrected for temperature and pressure. Each chamber has a volume of 0.08 cm^3^, diameter of 4.5 mm, and height of 5 mm. The spacing between adjacent chambers is 7.62 mm. The effective point of measurement is 3.6 mm from the surface [14].

### Preparing pre-treatment verification

**Figure 2:**
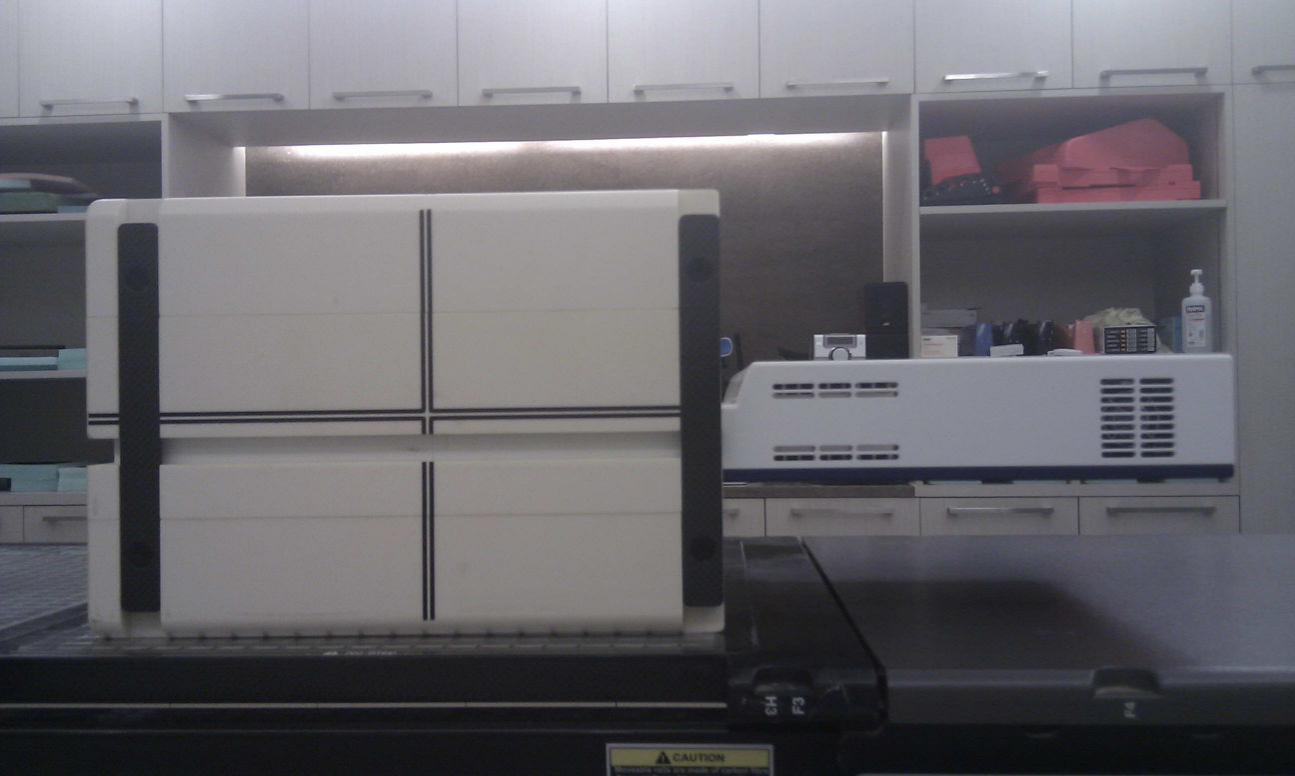
I’mRT MatriXX combined with the Multicube Phantom.

**Figure 3:**
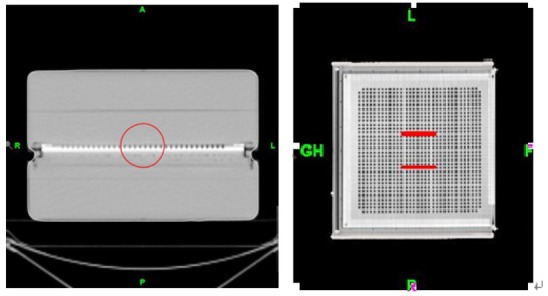
Cylindrical target outlined on the I’mRT MatriXX.

Images for coronal orientation of the MatriXX inserted into the Multicube Phantom (Figure 2) were scanned on a GE Light Speed RT 16 simulator. The scanned computed tomography (CT) images of the combined phantom were then imported into the Tomotherapy planning system. After choosing a correct image value-to-density table for the imported CT images, a cylindrical target, with a radius of 3.5 cm and length of 7 cm, was drawn on the CT images as shown in Figure 3. All treatment plans created in the study were based on this CT image set and were optimised to yield a dose of 2 Gy to the cylindrical target. Planning parameters of 2.5 cm field width, modulation factor 2.0, and fine calculation grid were used.

**Figure 4:**
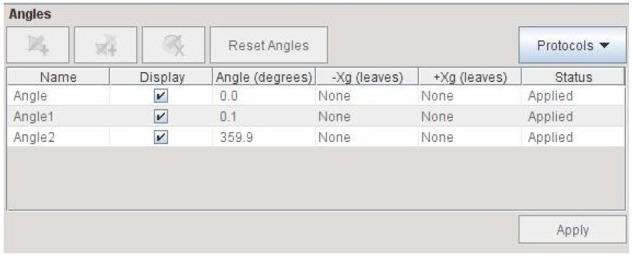
Beams setting simulating static beam at gantry 0°.

**Figure 5:**
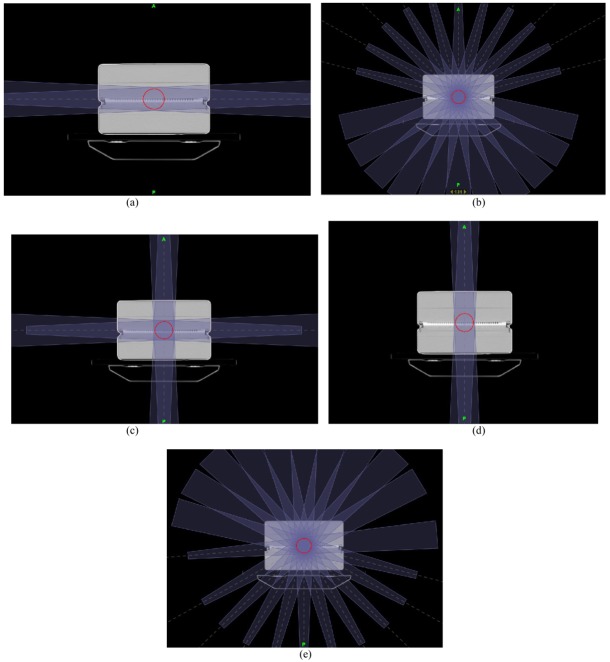
Different treatment plans for pre-treatment verification study. (a) Lateral opposing treatment plan (Gantry 90° and 270°). (b)Treatment plan with 11 fields irradiated from the front (Gantry 285°, 300°, 315°, 330°, 345°, 0°, 15°, 30°, 45°, 60°, and 75°). (c) 4-field box treatment plan (Gantry 0°, 90°, 180°, and 270°). (d) AP opposing treatment plan (Gantry 0° and 180°). (e) Treatment plan with 11 fields irradiated from the rear (Gantry 105°, 120°, 135°, 150°, 165°, 180°, 195°, 210°, 225°, 240°, and 255°).

Two types of treatment plans were studied:

(1) The first type was to evaluate the angular dependence of the I’mRT MatriXX by calculating the absolute dose measurement as a function of gantry angle of the TomoTherapy HD machine. Although TomoDirect mode allows radiation delivery at different gantry angles, TomoDirect planning does not support a plan calculation with one static treatment beam. To perform the study, three discrete treatment beams with gantry angle spread of ±0.1° were used instead to simulate a single static beam. When studying the output at gantry 0°, three treatment beams at gantry angles 359.9°, 0° and 0.1° were used (Figure 4). The plan was then optimised to yield a dose of at least 2 Gy to 95% volume of the cylindrical target. Finally, treatment verification was done on this plan using the MatriXX to analyse the accuracy of output measurement at gantry 0°. In total, 24 treatment plans were generated, with gantry angles ranging from 0° ± 0.1° to 345° ± 0.1° in 15° increments.

(2) The second type was to evaluate the effect of angular dependence of I’mRT MatriXX on the pre-treatment verification result. Five different TomoDirect treatment plans were created, including AP opposing (Figure 5(d)), lateral opposing (Figure 5(a)), 4-Field box (Figure 5(c)), a treatment plan with 11 fields irradiated from the rear (Figure 5(e)), and a treatment plan with 11 fields irradiated from the front (Figure 5(b)).

### TomoDirect treatment plan delivery

For the treatment plan delivery, the combined phantom was placed on the couch and aligned with the laser system, followed by an MVCT scan on the phantom using fine scan (2 mm) for the most precise alignment. Image fusion was then done on the planning CT and MVCT to ensure correct positioning of the phantom on the couch. Different verification plans were then irradiated. The 2D-array measurements were then analysed and compared with the dose plan imported from the Tomotherapy Treatment Planning System (TPS) in the OmniPro-I’mRT software. Dose comparisons in the high dose region (over 90% of prescription dose) were performed for first-type treatment plans while gamma tests with criteria 3 mm and 3% dose were performed for second-type treatment plans [17–18].

### Correction of measured dose due to angular dependence

**Figure 6:**
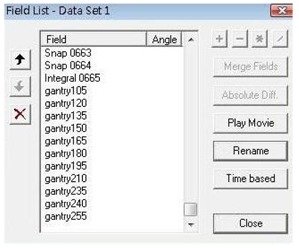
Snapshots taken by the MatriXX are merged into different fields according to the gantry angle.

**Figure 7:**
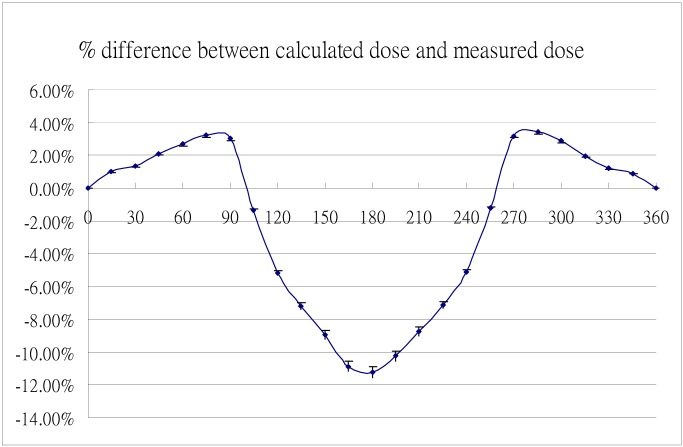
Percentage difference between measured dose and calculated dose at different gantry angles.

MatriXX angular dependence can be eliminated by applying the appropriate correction to the measured dose plane. The measured dose plane is the summation of the isodose snapshots taken in each second [8]. These snapshots were merged into different fields according to the gantry angle (Figure 6). The merged fields were then exported to the external software and corrected using the correction factors deviated from the dose measurement of simulated static treatment plans (Figure 7). After the correction, all processed fields were imported into the OmniPro-I’mRT and recombined into the new measured dose plane for dose difference analysis.

## Results

### Angular dependence

**Figure 8:**
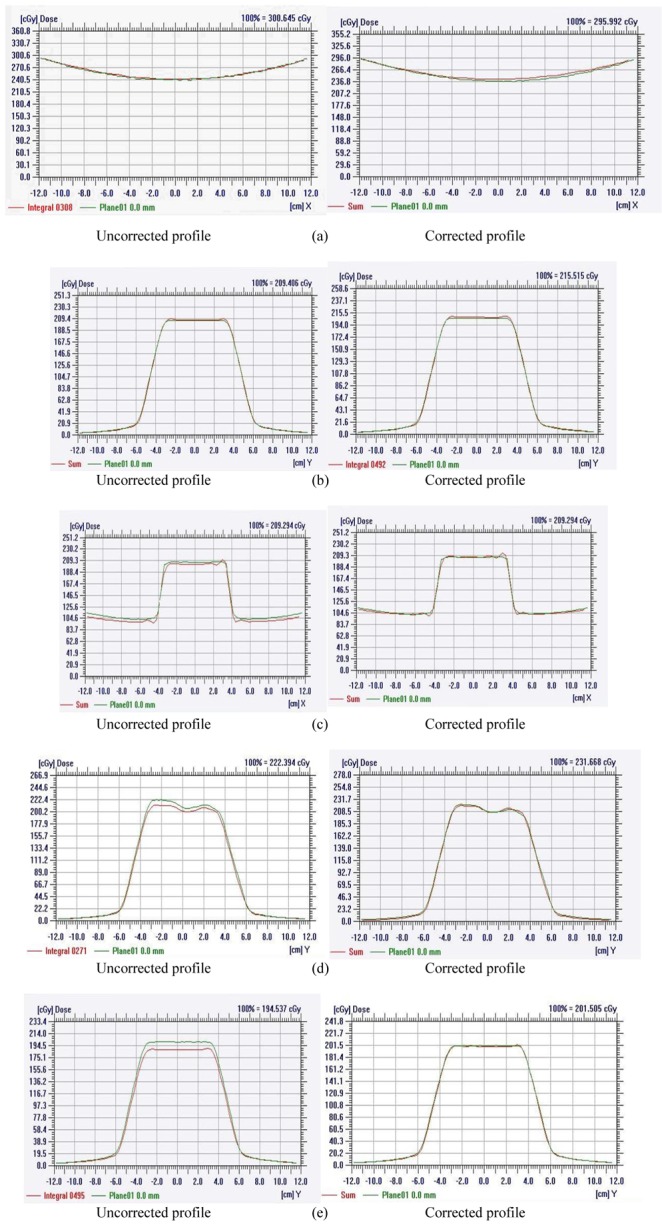
Comparison between the calculated dose profile (shown in green) and the measured dose profile (shown in red) with and without angular dependence correction for different treatment plans. (a) Lateral opposing treatment plan. (b) Treatment plan with 11 beams irradiated from the front. (c) 4-field box treatment plan. (d) AP opposing treatment plan. (e) Treatment plan with 11 beams irradiated from the rear.

Dose verifications for simulated static beam treatments with different irradiated directions were performed on the coronal plane containing the MatriXX detector. Figure 8 shows the average percentage difference between the calculated dose from the TPS and the measured dose with the MatriXX in the high dose region as a function of gantry angle. When the beam was incident on the front side of the MatriXX, the average percentage difference was within 3.5%. However, when the beam was irradiated from the rear, the dose deviation became apparent. The measured dose was between 6% and 11% lower than the calculated dose, with the maximum difference at gantry equal to 180°.

### Verification of different treatment plans without angular dependence correction

Table 1 summarises the result of gamma analysis on different treatment plan verifications. For treatment plans without dose contribution from the rear, such as lateral opposing and treatment plan with 11 beams irradiated from the front, the passing rate of gamma analysis (3%/3 mm) was more than 90%. For 4-field box treatment with about 25% rear dose contribution, the passing rate was less than 90%. For AP treatment, with about 50% rear dose contribution, the passing rate of gamma analysis further deteriorated to 79.5 %. The worst result was for the treatment plan with 11 beams irradiated from the rear. As all doses were contributed from the rear, the passing rate of gamma analysis was just 53.55%.

**Table 1:** Passing rate of gamma analysis in different treatment plan verifications without angular dependence correction.

**Treatment plan**	**Passing rate of gamma analysis (3%/3 mm)**
Lateral opposing	95.43%
11 treatment beams from the front	94.06%
4-field box	89.04%
AP opposing	79.50%
11 treatment beams from the rear	53.55%

### Verification of different treatment plans with angular dependence correction

Table 2 summarises the result of gamma analysis on different treatment plan verifications with angular dependence correction. The result is good as the passing rates of all treatment plans are all over 90% [18].

**Table 2:** Passing rate of gamma analysis in different treatment plan verifications with angular dependence correction.

**Treatment plan**	**Passing rate of gamma analysis (3%/3 mm)**
Lateral opposing	96.14%
11 treatment beams from the front	96.04%
4-field box	97.24%
AP opposing	91.28%
11 treatment beams from the rear	94.82%

## Discussion

The 2D-ion chamber array MatriXX shows angular dependence. This inherent property is due to the inhomogeneous effect at the air-high–Z material interface beneath the parallel plate chambers in the MatriXX [8], which introduces a large dose discrepancy for the posterior beams. When the MatriXX was used to verifiy TomoDirect treatment plans, the passing rate in gamma analysis could be very poor if no angular dependency correction was done to compensate for this inherent effect.

The TomoTherapy HD machine delivers radiation based on time rather than MU. The output of the machine therefore depends on its dose rate (MU/min). However, even with a well-calibrated TomoTherapy HD machine, there is a variation (±0.5%) in the delivered dose rate. Due to this fluctuation, it was not feasible to use a passing rate of 99% as a criterion for gamma test using the MatriXX, as achieved in RapidArc treatment plan verification [13]. Instead, a 90% passing rate was used [18].

## Conclusion

It is known that the I’mRT MatriXX 2D-array shows directional dependence. In Tomotherapy helical plan verification, this effect is diluted over the 360° gantry rotation, making it a suitable tool for pre-treatment verification. However, in TomoDirect treatment, the gantry is fixed at different discrete angles, causing a more obvious angular dependence effect. The difference between the measured dose with the I’mRT MatriXX and the TPS calculation can be as large as 12% for a treatment beam irradiated from the rear. In pre-treatment plan verification, the greater the dose contribution from the rear, the poorer the agreement between the measured dose and TPS. In conclusion, if the angular dependence of the I’mRT MatriXX is not corrected, a large dose discrepancy may be introduced to TomoDirect treatment plan verification results.
